# Effect of Soy Leaf Flavonoids on Pea Aphid Probing Behavior

**DOI:** 10.3390/insects12080756

**Published:** 2021-08-22

**Authors:** Katarzyna Stec, Bożena Kordan, Beata Gabryś

**Affiliations:** 1 Department of Botany and Ecology, University of Zielona Góra, Szafrana 1, 65-516 Zielona Góra, Poland; b.gabrys@wnb.uz.zgora.pl; 2 Department of Entomology, Phytopathology and Molecular Diagnostics, University of Warmia and Mazury in Olsztyn, Prawocheńskiego 17, 10-720 Olsztyn, Poland; bozena.kordan@uwm.edu.pl

**Keywords:** *Glycine max*, *Acyrthosiphon pisum*, electrical penetration graph, EPG, electropenetrography, apigenin, daidzein, genistein, kaempferol, herbivory, plant resistance

## Abstract

**Simple Summary:**

Flavonoids are plant phenolic compounds whose biological activities include participation in plant responses to various stresses of biological and environmental origins, including protection against insect herbivore attack. We were interested in whether the specific flavonoids detected in soybean leaves have the potential to discourage the pea aphid from infesting other leguminous plants, peas in particular. We immersed the pea leaves in ethanolic solutions of the flavonoids apigenin, daidzein, genistein and kaempferol, offered them to the pea aphids and observed their behavior when they probed plant tissues with their piercing–sucking mouthparts. We discovered that aphids readily probed the pea leaves whether they were treated with flavonoids or not. However, later on, the behavior of the aphids changed depending on the flavonoid applied. Apigenin, daidzein and kaempferol caused a decrease in the intensity of plant sap ingestion. In addition, daidzein and kaempferol made the finding of sap-transporting vessels more difficult for aphids. In contrast, genistein did not affect the pea aphids’ feeding activity. Our findings provide the plant breeders and plant protection services with information on what direction their efforts should take to protect leguminous plants against aphids in a sustainable and environmentally friendly way.

**Abstract:**

Flavonoids detected in soybean *Glycine max* (L.) Merr. (Fabaceae) cause various alterations in the metabolism, behavior, and development of insect herbivores. The pea aphid *Acyrthosiphon pisum* (Harris) (Hemiptera: Aphididae) poses potential threat to soybeans, but the effect of individual flavonoids on its feeding-associated behavior is relatively unknown. We monitored probing behavior (stylet penetration activities) of *A. pisum* on its preferred host plant, *Pisum sativum* L. untreated (control) and treated with 0.1% ethanolic solutions of flavonoids apigenin, daidzein, genistein, and kaempferol. We applied the electrical penetration graph (electropenetrography, EPG) technique, which visualizes the movements of aphid stylets within plant tissues. None of the applied flavonoids affected the propensity to probe the plants by *A. pisum.* However, apigenin enhanced the duration of probes in non-phloem tissues, which caused an increase in the frequency and duration of stylet mechanics derailment and xylem sap ingestion but limited the ingestion of phloem sap. Daidzein caused a delay in reaching phloem vessels and limited sap ingestion. Kaempferol caused a reduction in the frequency and duration of the phloem phase. Genistein did not affect aphid probing behavior. Our findings provide information for selective breeding programs of resistant plant cultivars to *A. pisum.*

## 1. Introduction

Soybean *Glycine max* (L.) Merr. (Fabaceae) flavonoids have been broadly studied for their importance in plant metabolism, the establishment of symbiotic relationships, response to abiotic and biotic stresses and human health-promoting effects [1,2,3,4,5,6,7]. Soybean is attacked by a number of insect pests, and some of them can cause severe economic damage. Soybean flavonoids have been identified as compounds that might negatively affect the behavior and development of herbivores. Specifically, a disruption in the metamorphosis of the redbanded stink bug *Piezodorus guildinii* (Westwood) (Hemiptera: Pentatomidae) occurred on certain genistein- and rutin-rich soybean cultivars [8]. Rutin reduced growth and deterred feeding of the cabbage looper *Trichoplusia ni* (Hübner) (Lepidoptera: Noctuidae) larvae and velvet bean *Anticarsia gemmatalis* Hübner (Lepidoptera: Noctuidae) caterpillars [9,10], and inhibited the early larval growth of the fruit-worm *Helicoverpa zea* (Boddie) (Lepidoptera: Noctuidae) [11]. Moreover, soybean flavonoids are induced by herbivory. Daidzein and formononetin in soybean leaves were induced by *Spodoptera litura* (F.) (Lepidoptera: Noctuidae) [3]. The infestation by soybean aphids *Aphis glycines* Matsumura (Hemiptera: Aphididae) caused the accumulation of daidzein, formononetin, and genistein [12]. Flavonoids were strongly induced in soybean leaves infested by the cowpea aphid *Aphis craccivora* Koch [13,14]. Likewise, an increase in flavonoid contents was observed in *Pisum sativum* L. (Fabaceae) infested by the pea aphid *Acyrthosiphon pisum* (Harris) (Hemiptera: Aphididae) [15,16,17]. The infestation of *A. pisum* induced the accumulation of genistein in alfalfa *Medicago sativa* L., which, in turn, reduced the survival rate of the aphids [18]. 

The natural induction of flavonoids in plants in response to insect feeding serves as a biomarker for the selection of resistant lines in breeding strategies [14]. The development of plant resistance has been considered as an alternative to neurotoxic insecticides for the purpose of preventing insect feeding [19]. Of the three plant resistance mechanisms, antixenosis, antibiosis, and tolerance, antixenosis and antibiosis are based on effects of plant traits on insect behavior and development. Tolerance is the ability of plants to survive insect damage [20]. Efforts, both conventional and using genetic engineering, have been made to obtain soybean lines and cultivars with at least moderate levels of antixenosis or antibiosis resistance to insects, including aphids [7,19,20,21]. The enhanced synthesis of flavonoids or the modification of their profiles in plants to make them less acceptable to herbivores are the suggested mechanisms for a biotechnological approach, as well as selective breeding programs in Fabaceae in general and in *G. max* in particular [14,22,23]. Interestingly, biopesticide synergy was observed when plant flavonoids were combined with entomopathogenic baculovirus: the soybean flavonoid compounds daidzein, genistein and kaempferol were found to synergistically improve biopesticide AfMNPV baculovirus activity against *T. ni* [24]. 

Flavonoids occur in many plant tissues, where they are present inside the cells or on the surfaces of different plant organs [22]. Herbivores such as Coleoptera or caterpillars of Lepidoptera and Hymenoptera (Symphyta) that possess chewing–biting mouthparts come into contact with flavonoids when they crush plant tissues for consumption. Following the damage, the plant structure is destroyed and allelochemicals are released from their storage spaces. The discharged flavonoids make contact with taste receptors on insect mouthparts and, depending on individual sensitivity and plant–herbivore compatibility, affect the herbivore behavior: they attract it to the plant or deter its feeding [25]. In contrast, herbivores such as Hemiptera or Thysanoptera that possess sucking–piercing mouthparts feed upon sap from various plant tissues without breaking plant integrity [26]. Among those herbivores, aphids (Hemiptera: Aphididae) exhibit the most sophisticated way of feeding: their mouthparts’ stylets penetrate plant tissues intercellularly to reach phloem sieve elements, from which they acquire nutrients [27]. Before they reach the phloem, aphids taste the contents of cells that are adjacent to the stylets’ route; stylets make short punctures into cells without destroying them and then aphids suck up samples of cell fluids [28]. At this stage, aphids may come into contact with plant allelochemicals, including flavonoids that are stored in epidermis and mesophyll cells [27,29]. When its stylets reach sieve elements, the aphid will possibly start feeding, i.e., it will uptake the phloem sap [27,30,31]. Along with nutrients, flavonoids transported through phloem vessels may be ingested [32,33,34]. Thus, plant flavonoids may affect aphid probing behavior when taken up from cells of peripheral tissues and/or sieve elements. In other words, the pre-ingestive and/or ingestive phases of probing, respectively, may be modified depending on the amount and/or the composition of flavonoid mixture in plant tissues.

The effect of individual flavonoids on the pea aphid probing behavior has rarely been investigated. Usually, flavonoids were added to sucrose–agarose diets [35,36,37]. The results of those experiments showed that high luteolin and genistein concentration reduced the ingestion of the diet by *A. pisum* [35]. At the same time, an increase in the developmental time, the pre-reproductive period, and mortality and a decrease in the fecundity and the intrinsic rate of natural increase in *A. pisum* occurred [35]. Naringenin reduced ingestion when applied in a low concentration and stimulated ingestion when applied in a high concentration [36]. Mixtures of saponins with apigenin incorporated into gels resulted in a reduction in the number of aphid probes and their duration [37]. A high concentration of genistein in artificial diets reduced the survival rate of the *Pisum* host race of *A. pisum* on *Medicago sativa* [18]. Under semi-natural conditions, when flavonoids were applied to the plant surface, rutin hindered the time it took to reach sieve elements and to accept phloem sap for continuous feeding by *A. pisum* on peas *Pisum sativum* L. (Fabaceae), while quercetin promoted probing activities of *A. pisum* within non-phloem and phloem tissues [38].

In our previous studies on antixenosis in eight soybean cultivars against the pea aphid, we discovered that *A. pisum* readily probed into leaf tissues of all cultivars, but the probes were usually terminated before they reached vascular tissues [39]. Nevertheless, the analyzed cultivars represented a spectrum of susceptibility to *A. pisum,* from relatively susceptible to highly resistant. We deduced that antixenosis mechanisms were active in peripheral tissues’ epidermis and/or mesophyll in soybean leaves, and we hypothesized that the varied response of *A. pisum* to individual cultivars was probably associated with the variation in soybean leaf flavonoid composition among these cultivars [39]. Apigenin and genistein occurred in all soybean cultivars, while the relatively susceptible or highly resistant cultivars also contained daidzein or kaempferol, respectively [39]. The content of apigenin ranged from 1.05 to 5.38 μg/g dry weight and genistein—from 0.61 to 3.05 μg/g dry weight, and the differences were not related to cultivar susceptibility levels [39]. We speculated at the time that the content of apigenin and genistein in all soybean cultivars studied probably made all of them relatively unacceptable to *A. pisum*, but the variation in susceptibility could have been caused by the presence of other flavonoids. The present study was designed to verify that idea.

The aim of the present work was to investigate the direct impact of flavonoids detected in soybean leaf tissues, specifically apigenin, daidzein, genistein and kaempferol, on pre-ingestive and ingestive phases of *A. pisum* probing on its host plant *P. sativum*. The effects of apigenin and genistein on *A. pisum* probing behavior were previously studied using artificial diets [35,37]. To our knowledge, the influence of daidzein and kaempferol on *A. pisum* probing behavior remains unknown. We decided to carry out our study under semi-natural conditions. For this purpose, we applied flavonoids to pea plants and monitored aphid probing using the electrical penetration graph (EPG) technique. EPG, known also as electropenetrography, visualizes the activities of aphid stylets in specific plant tissues. The values of parameters derived from EPG recordings are reliable and precise indicators of aphid behavioral responses to plant resistance mechanisms or modification in plant suitability due to the exogenous application of allelochemicals [40,41,42,43].

## 2. Materials and Methods

### 2.1. Plant and Aphid Cultures

The laboratory culture of *Pisum sativum*-derived *Acyrthosiphon pisum* was maintained as a multiclonal colony on *P. sativum* cv. “Milwa” in the laboratory at 20 °C, 65% r.h., and a L16:D8 photoperiod in a growing chamber, Sanyo MLR-351H (Sanyo Electronics Co. Ltd., Osaka, Japan). Two-to-three-day-old adult apterous females of *A. pisum* and three-week-old plants with two to three fully developed leaves were used for experiments. The plants used for experiments were the same plant species and cultivar that was used for the rearing of aphids. All experiments were carried out under the same conditions of temperature, r.h., and photoperiod as used for the rearing of plants and aphids.

### 2.2. Application of Flavonoids

**Figure 1:**
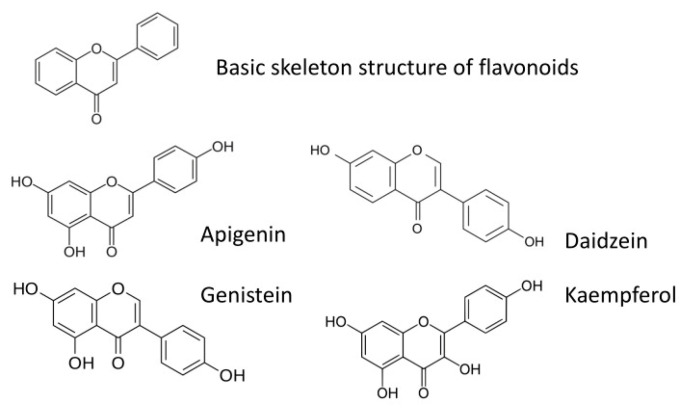
Basic skeleton structure of flavonoids and structures of flavonoids applied in the present study: apigenin, daidzein, genistein, and kaempferol.

Flavonoids apigenin, daidzein, genistein and kaempferol (Figure 1) were purchased from Sigma Aldrich, Poland. To mimic the natural environment under laboratory conditions, the flavonoids were offered to aphids by application through their host plants. The preparation and application of the compounds followed the procedure originally described by [44] with later modifications [38]. Briefly, each compound was dissolved in 70% ethanol to obtain the 0.1% solution. One leaf of an intact plant was dipped in the ethanolic solution of a given compound for 30 s, so all compounds were applied on the adaxial and abaxial leaf surfaces. Leaves of a similar size to the control plants were immersed in 70% ethanol, which was used as a solvent for the studied flavonoids. No negative effects of ethanol on plants or aphids were observed after it had been used according to the described procedure [42]. Our previous studies and works by other authors that used aphids as sensors demonstrated that exogenously applied compounds of various chemical groups, including flavonoids, penetrated the cuticle and epidermis and passed into deeper plant tissue layers. The transcuticular application of some of those compounds caused considerable disturbances in plant recognition and acceptance by aphids, which were reflected in the alterations of EPG-monitored aphid probing behavior [38,42,43,44]. The treated and control leaves were allowed to dry for 1 h before the start of the experiment to permit the evaporation of the solvent [38]. Every plant and aphid was used only once.

### 2.3. Monitoring of Aphid Probing Behavior (EPG No-Choice Test)

EPG requires the attachment of the plant and the aphid to their respective electrodes to make them parts of an electrical circuit. The circuit is completed when the aphid inserts its stylets into the plant. Weak voltage is supplied in the circuit, and all changing electric properties are recorded as EPG waveforms that can be correlated with aphid activities and the stylet position in plant tissues [27,40,45,46]. In the present study, aphids were connected to a golden wire electrode with conductive silver paint and starved for 1 h prior to the experiment. The probing behavior of *A. pisum* on peas untreated and treated with flavonoids was monitored for 8 h continuously using 4- and 8-channel DC EPG recording equipment (available at www.epgsystems.eu; Dillenburg 12, 6703 CJ Wageningen, the Netherlands). Signals were saved on the computer and analyzed with PROBE 3.1 software provided by dr. W. F. Tjallingii (available at www.epgsystems.eu); Dillenburg 12, 6703 CJ Wageningen, the Netherlands). The following aphid behaviors were distinguished: no penetration (waveform ‘np’—aphid stylets outside the plant), pathway phase penetration of non-phloem tissues (waveforms ‘A, B, and C’), derailed stylet movements (waveform ‘F’), salivation into sieve elements (waveform ‘E1’), ingestion of phloem sap (waveform ‘E2’), and ingestion of xylem sap (waveform ‘G’) [47]. Aphid activities visualized as EPG waveforms were analyzed according to their frequency and duration and presented in a configuration related to activities in peripheral and vascular tissues. The interpretation of EPG variables in terms of plant suitability to the aphids follows the interpretation provided by detailed studies on aphid–plant interactions [27,28,30,31,40,41,45,46,48,49].

Aphids for EPG experiments were 2–3-day-old (2–3 days after the final molt) viviparous apterous females selected randomly from the stock culture. According to [50], the use of aphids of random ages gives a clear view of the behavior of adult aphids within a population. The plants used in the bioassays were at growth stages 12–13 (two to three leaves unfolded) according to the BBCH scale (Biologische Bundesanstalt, Bundessortenamt und CHemische Industrie) [51]. Each aphid was given access to a freshly prepared plant. Each plant–aphid set was considered as a replication and was tested only once. The number of replications (=8 h EPG recordings) for each plant–flavonoid combination was 24 (i.e., two rounds of 12 simultaneous recordings). All experiments were carried out under the same conditions of temperature, relative humidity (r.h.), and photoperiod as those used for the rearing of plants and aphids. All bioassays started at 10:00–11:00 h MEST (Middle European Summer Time) and were carried out within five consecutive days for each round of EPG recordings (one day—12 replications of a given flavonoid treatment). Aphids show distinct diurnal feeding activity, with peak activity during daytime, independently of host plants [27,52].

### 2.4. Statistical Analysis

EPG variables describing aphid probing behavior were calculated using the Excel-VBA Macro [53], accessed from www.epgsystems.eu (accessed on 1 June 2021) and the means and standard deviations were subsequently calculated using the EPG analysis Excel worksheet created for this study [48,49]. In non-sequential variables (such as total durations of specific EPG waveforms and the number of EPG-recorded aphid activities), when a given waveform had not been recorded for an individual, the duration of that waveform was given the value of 0. In sequential variables (such as the time until specific EPG waveforms appeared in the recording), when variables related to the phloem phase (E1 or E2) were involved, only aphids that reached phloem phase were included in statistical analysis. The E1/E2 transition patterns were included in E2. The waveform patterns that were not terminated before the end of the experimental period (8 h) were included in the calculations. Recordings that terminated due to the aphid falling from the plant or where the EPG signal was unclear were discarded from analysis. Only the replications that included a complete 8 h recording were kept for analysis, which were: control (*n* = 23), apigenin-treated (*n =* 17), daidzein-treated (*n =* 18), genistein-treated (*n =* 16), kaempferol-treated (*n =* 15). Due to failure to meet the assumptions of analysis of variance, the obtained data were analyzed by the Kruskal–Wallis test and post hoc multiple comparisons of mean ranks for all groups (Dunn’s test). The Kruskal–Wallis test is a non-parametric alternative to the one-factor ANOVA test for independent measures and it is commonly used to analyze data deriving from EPG recordings of aphid probing. The mean and SD values given in Table 1 is a representation of non-Gaussian data, but the statistical analysis was carried out by non-parametric tests in which all individual data were included. All statistical calculations were performed using StatSoft (Statistica 13.3 package).

## 3. Results

**Figure 2:**
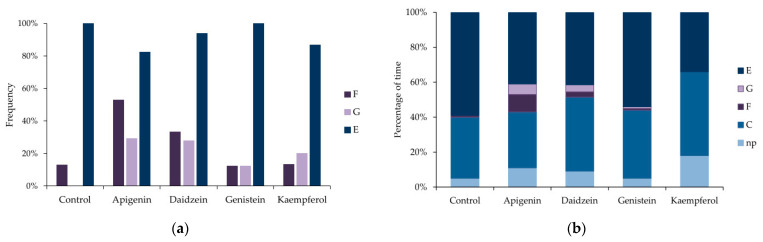
Occurrence of waveform patterns demonstrating EPG-recorded stylet penetration activities of *Acyrthosiphon pisum* on *Pisum sativum* non-treated (*n =* 23) and treated with 0.1% apigenin (*n =* 17), daidzein (*n =* 18), genistein (*n =* 16) and kaempferol (*n =* 15): (**a**) frequency of occurrence of E (phloem phase), G (xylem phase), and F (derailed stylet activities) expressed as percentage of aphids that showed given activity; (**b**) percentage of time assigned to non-probing ‘np’ and probing activities (pathway C, activity F, xylem phase G and phloem phase E = phloem salivation E1 + phloem sap ingestion E2) Figure 2b is based on data presented in Table 1.

EPG waveforms generated at the aphid–plant interface visualized three major aphid activities, irrespective of treatment: non-probing (=aphid stylets outside the plant), probing in non-vascular tissues epidermis and mesophyll, and probing in vascular tissues xylem and phloem (Table 1, Figure 2a,b).

Probing in non-vascular tissues included typical pathway activity that represents the progressive movement of stylets within the apoplast accompanied by short punctures into cells adjacent to the stylet track (EPG waveform C) and derailed stylet activities (EPG waveform F). Probing in vascular tissues embraced the ingestion of xylem sap (EPG waveform G), salivation into sieve elements (EPG waveform E1), and ingestion of phloem sap from sieve elements (EPG waveform E2) (Table 1, Figure 2a,b).

**Figure 3:**
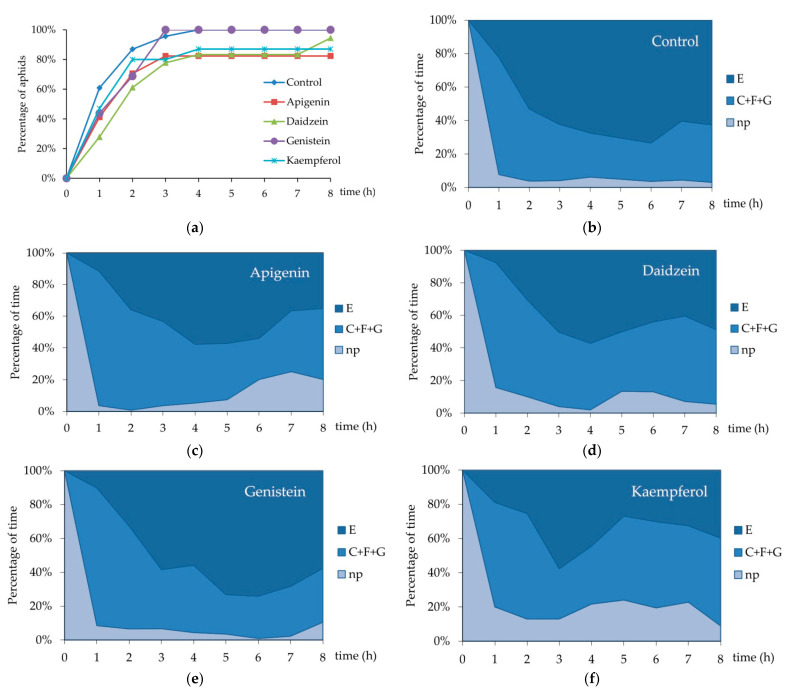
Sequential changes in EPG-recorded stylet penetration activities of *Acyrthosiphon pisum* on *Pisum sativum*: (**a**) Cumulative percentage of aphids that attained phloem phase during the 8 h EPG monitoring on *P. sativum* treated with 0.1% apigenin, daidzein, genistein and kaempferol; (**b**) sequential changes in penetration activities of *A. pisum* on control untreated *P. sativum*; (**c**) sequential changes in penetration activities of *A. pisum* on *P. sativum* treated with 0.1% apigenin; (**d**) sequential changes in penetration activities of *A. pisum* on *P. sativum* treated with 0.1% daidzein; (**e**) sequential changes in penetration activities of *A. pisum* on *P. sativum* treated with 0.1% genistein; (**f**) sequential changes in penetration activities of *A. pisum* on *P. sativum* treated with 0.1% kaempferol. Panels (**b**–**f**) represent the proportion of time (average percentage of cumulative time for aphids in the group) devoted to np—non-probing, C + F + G—pathway + derailed stylet activities + xylem phase, and E—phloem phase E1 (salivation) + E2 (sap ingestion) activities of *A. pisum* during the consecutive hours of 8 h EPG recording (control *n =* 23; apigenin-treated *n =* 17; daidzein-treated *n =* 18; genistein-treated *n =* 16; kaempferol-treated *n =* 15).

On the control plants, aphid stylet penetration within plant tissues (= total duration of probing) occupied 95% of the 8 h aphid access time to plants (Table 1). All aphids showed pathway activity C and phloem phase E, while derailed stylet activities F occurred in 13% of aphids and no aphid showed xylem phase G (Figure 2a). Aphids devoted 5% of the experimental time to non-probing activities, 35% to typical pathway probing C, derailed stylet activities F occupied 1% of time on average, and phloem phase E, 59% (Figure 2b). Phloem phases E1 and E2 engaged 62% of total probing time (phloem phase index = 0.62 ± 0.04) (Table 1). Probing was divided into 16.5 ± 2.0 probes of 0.7 h duration on average. Brief, shorter than 3 min probes amounted to 51% of all probes. The first probe was usually made 1.3 min after aphids gained access to the plant and was 30.6 ± 12.2 min long on average. Nearly 30% of all probes and 38% of brief probes occurred before the first phloem phase. The first phloem phase appeared 1.0 ± 0.2 h after aphids started probing and it usually comprised sustained sap ingestion (Table 1). All aphids reached sieve elements (= started phloem phase) within three hours from the onset of the experiment (Figure 3a) and activities within the phloem were the main aphid occupation from the second hour of the experiment onwards (53–73% of all activities) (Figure 3b). Sap ingestion occurred in 4.6 periods on average, and each was approximately 1.4 h long. The participation of salivation in phloem phase activities was relatively low (phloem salivation index = 0.04 ± 0.01) (Table 1).

On peas treated with 0.1% apigenin, 11% of the time was assigned to non-probing, 32% to pathway C, and 41% to the phloem phase. Activities F and G did occur in aphids on apigenin-treated plants and their frequencies and durations were relatively high; activity F occurred in 53% of aphids and occupied 10% of the time, while G occurred in 29% of aphids and engaged 6% of the time (Table 1, Figure 2a,b). Statistically significant differences with respect to the control were detected in the number and the mean duration of all probes and probes before the first phloem phase (Table 1; Kruskal–Wallis test, *p* < 0.05). There were 2.5 times fewer probes in general and 2.8 times fewer probes before the first phloem phase on apigenin-treated peas than on the control. Accordingly, the probes were 2.3 times longer than on the control peas (Table 1). The proportion of phloem phase during probing was similar to the control, but only 80% of aphids reached the phloem phase within 8 h of access to plants (Figure 3a). In addition, the phloem phase never exceeded 60% of aphid activities on plants and the proportion of sap ingestion activity decreased over time to reach 35% of all activities at the end of the monitoring period (Figure 3c).

On peas treated with 0.1% daidzein, 9% of the time was assigned to non-probing, 42% to pathway C, and 41% to the phloem phase. Activity F occurred in 33% of aphids and occupied 3% of the time, while G occurred in 28% of aphids and engaged 4% of the time (Table 1, Figure 2a,b). Aphids made significantly fewer brief probes than on the control: there were 1.5 fewer brief probes in general (Table 1; Kruskal–Wallis test; *p* < 0.05), but the number of brief probes before the first phloem phase was similar as compared to the control (Table 1). Although nearly all aphids reached the phloem phase (Figure 3a), they needed almost three times more time to reach the sieve elements than on the control. At the same time, the number of phloem phases was significantly lower in aphids on daidzein-treated plants than in aphids on the control peas (Table 1). Generally, the proportion of time assigned to sap ingestion was slightly lower than in the control and never exceeded 60% of all aphid activities on plants (49% at the end of the monitoring period) (Figure 3d).

On peas treated with 0.1% genistein, 11% of the time was assigned to non-probing, 32% to pathway C, and 41% to the phloem phase. The frequency and duration of F and G were relatively low: F and G occurred in 13% of aphids and each engaged 1% of the time (Table 1, Figure 2a,b). Aphid behavior was generally similar to that on the control. However, the number of phloem sap ingestion phases was 2.4 times lower than on the control (Table 1). All aphids reached the sieve elements within three hours after access to the plants (Figure 3a) and the proportion of time assigned to sap ingestion was relatively high (within the range of 10–75% of all activities) and comparable to the control during the 8 h of monitoring (58% at the end of monitoring period) (Figure 3e).

On peas treated with 0.1% kaempferol, 18% of the time was assigned to non-probing, 48% to pathway C, and 34% to the phloem phase. Activities F and G did not occur (Table 1, Figure 2a,b). The total time of non-probing was 3.5 times longer than on the control; non-probing intervals between probes were significantly longer than on control (Table 1; Kruskal–Wallis test, *p* < 0.05). Non-probing occupied 10% to 25% of all aphid activities during the monitoring period (Figure 3f). Within eight hours after access to plants, 87% of aphids managed to reach the sieve elements (Figure 3a) but the proportion of time assigned to sap ingestion ranged from 19% to 58% of all activities (40% at the end of monitoring period) (Figure 3f). The phloem phase index was relatively low (0.37 ± 0.08 as compared to 0.62 ± 0.04 on control) (Table 1).

## 4. Discussion

The specificity of aphid response to non-volatile plant allelochemicals derives from the lack of contact chemoreceptors on their mouthparts. In contrast to biting–chewing insects that possess chemosensitive sensilla on various elements of the feeding apparatus and in the wall of cibarium, the fluid-feeding herbivores, including aphids, have no chemoreceptors on the stylets that enter the tissues of the host [54]. The only taste organ is located in the hypopharynx [27,55,56]. The existence and activity of external contact chemoreceptors on the aphid body, such as sensilla at the tibial–tarsal junction, have not been confirmed unambiguously, although it was shown that aphids could detect non-volatile chemicals on plant surface using the hairs on the tips of antennae [27]. Thus, once an aphid finds itself on a plant, it must insert its stylets into plant tissues to gather and evaluate information on the suitability of the substrate [56]. Aphids usually start probing immediately after they are allowed to, both under natural and laboratory conditions [57]. The collection of chemical information by the probing aphid is a two-stage process. First, during the pre-ingestive stage, the aphid probes within peripheral tissues and tests small cytoplasm samples from cells adhering to stylet tracks [28]. Then, if the probing continues and the stylets reach phloem tissues, the aphid switches to the ingestive stage and starts the uptake of sap from sieve elements [58]. The continuation of probing both in peripheral and phloem tissues depends on the composition of allelochemicals and the allelochemical/nutrient proportion in these tissues, respectively [41,56,59,60]. The termination of probing during the pre-ingestive stage may indicate the presence of feeding deterrents in peripheral tissues, while the termination of sap ingestion soon after the onset of feeding may indicate either the presence of feeding deterrents or low nutritional value of sap, or both [56,59,61]. 

We discovered several important differences in the pea aphid behavior on flavonoid-treated peas in relation to the control. These differences occurred in diverse probing phases, depending on the flavonoid treatment. The behavior of aphids at the plant surface, before they inserted stylets in experimental plants for the first time, was rather consistent. There was no delay in the onset of probing on plants treated with flavonoids in relation to the control. This means that aphids did not respond to deterrent factors, if they existed, at this stage of the probing process. However, the time to initiate a probe was the shortest on apigenin-treated plants in relation to other flavonoid treatments. Nevertheless, it is typical for aphids to start probing in a few minutes’ time after access to their host plants if no repellent factors are present [62,63,64,65]. The initiation of stylet penetration by aphids in EPG experiments may be affected by a variety of internal and external factors, such as plant surface features including color, texture and phytochemicals (volatile and non-volatile) [62]. In addition to wax and cutin, the plant cuticle contains secondary metabolites, including flavonoids [66,67]. Aphids can detect non-volatile chemicals on the plant surface by using contact chemoreceptors on the tips of antennae [68]. Aphids touch plants with antennae before probing and when they insert stylets, they position their antennae along the body [27,68,69]. We cannot exclude the fact that a certain amount of exogenously applied flavonoids in the present study remained on the plant surface, but apparently it did not affect aphid behavior significantly. A similar lack of effect on the time taken to initiate the first penetration was observed in the EPG-recorded behavior of the peach potato aphid *Myzus persicae* (Sulz.) on plants treated with deterrent polygodial [70], nerolidol and farnesol [71], or other compounds [38,42,43,63,68,69]. In summary, we determined that none of the applied flavonoids affected the propensity to probe the plants by *A. pisum*, as all aphids on all plants started the first probe almost immediately after having access to the leaves.

The insertion of stylets in plant tissues begins the pre-ingestive stage in aphid probing. The period before the first period of activity in phloem tissues from the onset of the experiment (time to first E1 in experiment from first probe) depends on epidermal, mesophyll, general vascular, and early phloem factors. The first cells that are punctured for gustatory purposes are epidermal and outer mesophyll cells [28]. The time needed by stylets to cross one layer of cells is approximately 2–3 min [72]; therefore, a high proportion of probes shorter than that time, especially during the phase before phloem elements are reached, suggests the presence of deterrent factors in outer plant tissues, mainly the epidermis [58,59,73,74]. On non-host plants, resistant plant cultivars or plants treated with feeding deterrents, the total duration of probing, the duration of the first probe, the duration of all probes and especially probes that precede the first phloem phase are good indicators of plant suitability and the values of these parameters are usually lower than on suitable hosts [27,45,58,75]. In the present study, the total duration of probing was similar on all plants, except kaempferol-treated plants, on which an increase in the total duration of non-probing and the mean duration of non-probing intervals between probes occurred. During the pre-ingestive stage, when aphid stylets probed within non-vascular tissues, all aphids spent similar time on typical pathway activity C, which was the progressive movement of stylets towards the phloem. At the same time, the total number of probes and the number of brief probes shorter than 3 min was lowest on apigenin-treated plants and highest on kaempferol-treated plants. On apigenin-treated plants, aphids needed fewer but longer probes before the phloem phase than on other plants, including the control. However, an increase in the frequency and duration of derailed stylet mechanics visualized as waveform F occurred on apigenin- and daidzein-treated plants. The occurrence of waveform F in EPG recordings is difficult to interpret. It is usually attributed to mechanical stylet difficulties when for some reason stylets lose their proper position in the stylet bundle and therefore are unable to penetrate normally [76]. The increased frequency and duration of F was observed in aphids on resistant plants [77], ageing plants [73], insecticide-treated plants [78], on virus-infected plants (*M. persicae* on cabbage) [79], in aposymbiotic aphids [73,80] and in parasitized aphids [81]. On the other hand, fewer instances of F occurred in aphids whose structural sheath protein was silenced [82] or on plants with *Potato Leafroll Virus* (PLRV) infection symptoms (*M. persicae* on potato) [73].

The time taken to reach the first sieve element phase is a good indicator of deterrent factors that prevent the aphid from the continuation of pathway probing towards vascular tissues [83]. Indeed, the time taken to reach the first sieve element phase on resistant plant genotypes was significantly greater than in susceptible genotypes [84,85,86]. Similar delays before the first phloem phase were observed in the pea aphid after the exogenous application of rutin [38], and in peach potato aphid after the application of naringenin derivatives [87], citral and its derivatives [88], β-thujone oxime [71], cis-jasmone [43], or nerolidol [83]. In the present study, considerable delay in finding phloem tissues and commencing sap ingestion occurred on daidzein-treated plants in comparison to control and other flavonoid-treated plants. The highest success rate in reaching phloem vessels occurred on genistein-treated plants and the lowest on apigenin and kaempferol-treated plants.

The ingestive stage in pea aphid probing on flavonoid-treated plants in the present study consisted of bouts of xylem and phloem sap ingestion. On apigenin- and daidzein-treated plants, xylem sap ingestion occurred relatively more frequently and lasted longer than on other flavonoid-treated plants. On the control plants, xylem sap ingestion did not occur. Xylem sap ingestion is generally considered an aphid strategy for maintaining or restoring water (osmotic) balance [89,90,91]. However, the incidence of this activity may also be related to aphid age, plant genotype, and their interaction [59] or the abundance of obligatory symbionts [90]. Nevertheless, phloem sap is a basic food source of aphids [61]. In the present study, the highest proportion of phloem phase and phloem sap ingestion phase in all probing activities occurred on genistein-treated plants. Additionally, on genistein-treated plants, the individual bouts of sap ingestion activity were longest. In contrast, a visible trend towards the reduction in phloem phase duration occurred on kaempferol-treated plants. The shorter duration of sap ingestion in resistant genotypes or non-hosts shows that resistance factors are present in phloem tissues [58,84,85,86,92,93,94]. The reduction in the duration of sap ingestion bouts may also be caused by the exogenous application of xenobiotics, such as halogenated piperitone derivatives [95], farnesol and nerolidol [83] or camphene and ionone [88]. Sap ingestion activity is always preceded by a short phase of salivation to prevent the phloem wound response [96,97]. Repeated and prolonged phloem salivation is common on resistant plants or non-hosts and indicates the presence of antixenosis factors in phloem vessels [92,96,98,99,100]. The proportion of salivation during the phloem phase was similar in all aphids in the present study, irrespective of treatment.

The results of EPG experiments demonstrated a wide spectrum of changes in the pea aphid probing behavior induced by the exogenous application of selected flavonoids to pea plants. None of the flavonoids prevented *A. pisum* from probing. The activity of apigenin was expressed in non-vascular and vascular tissues: apigenin enhanced the duration of probes in non-phloem tissues and caused an increase in the frequency and duration of stylet mechanics derailment and xylem sap ingestion but deterred the ingestion of phloem sap. The activity of daidzein was detectable in non-vascular and vascular tissues: daidzein caused a delay in reaching phloem vessels and moderately limited sap ingestion. Genistein did not affect aphid probing behavior. Kaempferol caused a reduction in the frequency and duration of the phloem phase. 

In our former study, we hypothesized that the content of apigenin and genistein in the eight soybean cultivars examined probably made them relatively unacceptable to the pea biotype of *A. pisum* [39]. The outcomes of the present work partly support our deductions. Apparently, genistein is very well tolerated by the pea aphid, which we demonstrated in our study, and which is in accordance with data derived in experiments with the use of artificial diets: genistein caused negative effects on the probing behavior and life parameters of *A. pisum* only at high concentrations [18,35]. At the same time, soybean cultivar ‘Aldana’, which had the lowest content of genistein, was the most resistant cultivar [39]. Therefore, we are of the opinion that an increase in genistein in plant tissues would not necessarily make them more resistant to *A. pisum*. However, it is likely that the enrichment of plants with apigenin may be the right direction for breeding resistant cultivars. The long duration of non-probing intervals between individual probes may suggest that aphids might have probably walked out from the plants, had they been free to move. At the same time, apigenin restricted sap ingestion on the treated plants, which confirms results of studies that applied artificial diets [37]. Our current findings also seem to explain the riddle of two soybean cultivars ‘Madlen’ and ‘Violetta’ which had similar flavonoid composition: both contained genistein, apigenin and daidzein but ‘Madlen’ was relatively susceptible, while ‘Violetta’ was relatively resistant [39]. The content of genistein was three times higher in ‘Violetta’ than in ‘Madlen’ but, as stated earlier, it is unlikely that genistein was of major importance. The content of daidzein was 2.3 times higher in relatively susceptible ‘Madlen’ than in ‘Violetta’ while the content of apigenin was 4.7 times lower in ‘Madlen’. Both daidzein and apigenin appear to restrict the pea aphid probing activities, but the relatively higher content of apigenin probably makes ‘Violetta’ less palatable to *A. pisum* than ‘Madlen’. Finally, it can be presumed that the total unacceptability of cultivar ‘Aldana’ to *A. pisum* is caused by apigenin, genistein, kaempferol and rutin. However, the content of apigenin was comparable to the contents of this flavonoid in relatively acceptable soybean cultivars and the content of genistein was one of the lowest. In another study, we determined that rutin caused a delay in reaching phloem vessels and a reduction in the duration of sap ingestion [38]. However, the content of rutin in this cultivar was 4.7 times lower than in the relatively susceptible cultivar ‘Augusta’. We suggested at the time that kaempferol was probably responsible for the rejection of ‘Aldana’ by *A. pisum*. The present study confirmed that idea. 

In summary, the application of apigenin and daidzein generates modifications in the pre-ingestive and ingestive stages of probing of *A. pisum* and the application of kaempferol affects the ingestive stage of the pea aphid probing activities. However, additional experiments will be needed to establish the optimal dose and the concentration and persistence of exogenously applied flavonoids in plant tissues if these compounds were considered for practical use, as the effects of flavonoid treatments are clearly dose-dependent [36,37,38].

## Figures and Tables

**Table 1:** EPG-recorded stylet penetration activities of *Acyrthosiphon pisum* on *Pisum sativum* treated with 0.1% ethanolic solutions of apigenin, daidzein, genistein and kaempferol. All values represent means ± SD of cumulative time or number of events within an 8 h period. Different letters in rows denote statistically significant differences among treatments (Kruskal–Wallis test, *p* < 0.05).

EPG Variable ^1^		Control	Apigenin	Daidzein	Genistein	Kaempferol
*General aspects*		*n =* 23	*n =* 17	*n =* 18	*n =* 16	*n =* 15
Total duration of non-probing ^2^	h	0.4 ± 0.1 ab	0.9 ± 0.3 a	0.7 ± 0.2 ab	0.4 ± 0.1 ab	1.4 ± 0.4 b
Total duration of probing ^2^	h	7.6 ± 0.1 ab	7.1 ± 0.3 a	7.3 ± 0.2 ab	7.6 ± 0.1 ab	6.6 ± 0.4 b
Total duration of pathway (C) ^2^	h	2.8 ± 0.2 a	2.5 ± 0.3 a	3.4 ± 0.4 a	3.1 ± 0.4 a	3.8 ± 0.4 a
Total duration of derailed stylet activities (F) ^2^	min	6.4 ± 3.9 a	50.1 ± 16.3 a	16.6 ± 7.1 a	4.8 ± 3.4 a	0.1 ± 0.0 a
Total duration of xylem phase (G) ^2^	min	0.0 ± 0.0 a	28.1 ± 15.7 a	20.9 ± 10.4 a	6.9 ± 4.7 a	1.5 ± 0.8 a
Total duration of pathway C+F+G ^2^	h	2.9 ± 0.3 a	3.8 ± 0.3 a	4.0 ± 0.4 a	3.3 ± 0.4 a	3.8 ± 0.4 a
Total duration of phloem phase E1+E2 ^2^	h	4.7 ± 0.3 a	3.3 ± 0.5 a	3.3 ± 0.5 a	4.3 ± 0.5 a	2.7 ± 0.6 a
Total duration of phloem sap ingestion phase E2 ^2^	h	4.6 ± 0.3 a	3.2 ± 0.5 a	3.2 ± 0.5 a	4.3 ± 0.5 a	2.7 ± 0.6 a
Phloem phase index (E1+E2)/(C+E1+E2+G+F) ^2^		0.62 ± 0.04 a	0.42 ± 0.07 a	0.43 ± 0.07 a	0.56 ± 0.06 a	0.37 ± 0.08 a
Number of probes ^2^	#	16.5 ± 2.0 b	6.5 ± 0.9 a	12.4 ± 1.5 ab	11.1 ± 1.5 ab	22.8 ± 4.0 b
Number of brief probes <180 s ^2^	#	8.4 ± 1.4 b	1.5 ± 0.4 a	5.6 ± 1.0 b	4.8 ± 1.0 ab	12.1 ± 2.5 b
Mean duration of a probe ^2^	h	0.7 ± 0.2 b	1.7 ± 0.4 a	0.7 ± 0.1 ab	0.9 ± 0.1 ab	0.6 ± 0.2 b
Mean duration of np intervals ^2^	min	1.4 ± 0.1 a	9.3 ± 4.2 ab	3.2 ± 0.7 ab	2.5 ± 0.5 ab	3.5 ± 0.7 b
Time to first probe ^2^	min	1.3 ± 0.4 ab	0.3 ± 0.1 a	1.8 ± 0.3 b	1.6 ± 0.7 b	1.4 ± 0.3 b
Duration of first probe ^2^	min	30.6 ± 12.2 a	80.3 ± 30.5 a	14.5 ± 8.3 a	23.3 ± 8.2 a	13.8 ± 7.5 a
Probing in non-phloem tissues before phloem phase						
Number of probes before first phloem phase ^3^	#	*n =* 23 4.8 ± 1.0 ab	*n =* 14 1.7 ± 0.3 b	*n =* 17 5.6 ± 1.2 a	*n =* 16 4.2 ± 0.7 ab	*n =* 13 5.8 ± 2.2 ab
Number of brief probes before first phloem phase ^3^	#	*n =* 23 3.2 ± 0.7 b	*n =* 14 0.7 ± 0.3 a	*n =* 17 2.6 ± 0.5 b	*n =* 16 2.3 ± 0.4 ab	*n =* 13 3.8 ± 1.5 ab
Time from first probe to first phloem phase E1 ^3^	h	*n =* 23 1.0 ± 0.2 a	*n =* 14 1.2 ± 0.2 a	*n =* 17 2.2 ± 0.5 a	*n =* 16 1.6 ± 0.2 a	*n =* 13 1.1 ± 0.3 a
Time from first probe to first phloem phase E1+E2 ^3^	h	*n =* 23 1.0 ± 0.2 b	*n =* 14 1.3 ± 0.2 ab	*n =* 17 2.9 ± 0.7 a	*n =* 16 1.6 ± 0.2 ab	*n =* 13 1.1 ± 0.3 ab
Time from first probe to first sustained sap ingestion phase (E2 > 10 min) ^5^	h	*n =* 23 1.1 ± 0.2 b	*n =* 14 1.4 ± 0.2 ab	*n =* 17 2.7 ± 0.6 a	*n =* 16 1.7 ± 0.2 ab	*n =* 13 1.4 ± 0.3 ab
Probing in phloem tissues		*n =* 23	*n =* 14	*n =* 17	*n =* 16	*n =* 13
Number of phloem phases E1+E2 ^3^	#	4.6 ± 0.5 b	2.9 ± 0.3 ab	2.8 ± 0.6 a	2.5 ± 0.3 ab	2.8 ± 0.4 ab
Mean duration of phloem phase E1 + E2 ^4^	h	1.6 ± 0.6 a	1.8 ± 0.5 a	2.1 ± 0.4 a	2.2 ± 0.4 a	1.4 ± 0.5 a
Mean duration of phloem sap ingestion phase E2 ^4^	h	1.4 ± 0.4 a	1.3 ± 0.5 a	2.1 ± 0.5 a	2.2 ± 0.4 a	1.4 ± 0.5 a
Phloem salivation index E1/(E1 + E2) ^3^		0.04 ± 0.01 a	0.03 ± 0.01 a	0.03 ± 0.01 a	0.02 ± 0.01 a	0.05 ± 0.02 a

^1^ C = pathway, F = derailed stylet activities, G = xylem sap ingestion, E1 = watery salivation into sieve elements, E2 = phloem sap ingestion, np = non-probing; ^2^ all replicates (= individual EPG recordings) were included in statistical analysis irrespective of the presence of phloem phase; ^3^ only replicates that embraced at least phloem phase E1 were included in the statistical analysis; ^4^ only replicates that embraced phloem phase E1 and E2 were included in analysis; ^5^ only replicates that embraced phloem sap ingestion phase E2 > 10 min were included in analysis; *n =* number of replicates included in statistical analysis; # = number of EPG events.

## Data Availability

All data are contained in the present article.
